# COVID-19 Disrupted Provision and Utilization of Health and Nutrition Services in Uttar Pradesh, India: Insights from Service Providers, Household Phone Surveys, and Administrative Data

**DOI:** 10.1093/jn/nxab135

**Published:** 2021-06-03

**Authors:** Phuong H Nguyen, Shivani Kachwaha, Anjali Pant, Lan M Tran, Monika Walia, Sebanti Ghosh, Praveen K Sharma, Jessica Escobar-Alegria, Edward A Frongillo, Purnima Menon, Rasmi Avula

**Affiliations:** Poverty, Health and Nutrition Division, International Food Policy Research Institute, Washington, DC, USA; Poverty, Health and Nutrition Division, International Food Policy Research Institute , New Delhi, India; Poverty, Health and Nutrition Division, International Food Policy Research Institute , New Delhi, India; FHI Solutions, Hanoi, Vietnam; Poverty, Health and Nutrition Division, International Food Policy Research Institute, New Delhi, India; FHI Solutions, New Delhi, India; FHI Solutions, New Delhi, India; FHI Solutions, Washington, DC, USA; Health Promotion, Education, and Behavior, Arnold School of Public Health, University of South Carolina , Columbia, SC, USA; Poverty, Health and Nutrition Division, International Food Policy Research Institute , New Delhi, India; Poverty, Health and Nutrition Division, International Food Policy Research Institute , New Delhi, India

**Keywords:** COVID-19, service delivery, service utilization, nutrition, India

## Abstract

**Background:**

The coronavirus (COVID-19) pandemic may substantially affect health systems, but little primary evidence is available on disruption of health and nutrition services.

**Objectives:**

This study aimed to *1*) determine the extent of disruption in provision and utilization of health and nutrition services induced by the pandemic in Uttar Pradesh, India; and *2*) identify how adaptations were made to restore service provision in response to the pandemic.

**Methods:**

We conducted longitudinal surveys with frontline workers (FLWs, *n* = 313) and mothers of children <2 y old (*n* = 659) in December 2019 (in-person) and July 2020 (by phone). We also interviewed block-level managers and obtained administrative data. We examined changes in service provision and utilization using Wilcoxon matched-pairs signed-rank tests.

**Results:**

Compared with prepandemic, service provision reduced substantially during lockdown (83–98 percentage points, pp), except for home visits and take-home rations (∼30%). Most FLWs (68%–90%) restored service provision in July 2020, except for immunization and hot cooked meals (<10%). Administrative data showed similar patterns of disruption and restoration. FLW fears, increased workload, inadequate personal protective equipment (PPE), and manpower shortages challenged service provision. Key adaptations made to provide services were delivering services to beneficiary homes (∼40%–90%), social distancing (80%), and using PPE (40%–50%) and telephones for communication (∼20%). On the demand side, service utilization reduced substantially (40–80 pp) during the lockdown, but about half of mothers received home visits and food supplementation. Utilization for most services did not improve after the lockdown, bearing the challenges of limited travel (30%), nonavailability of services (26%), and fear of catching the virus when leaving the house (22%) or meeting service providers (14%).

**Conclusions:**

COVID-19 disrupted the provision and use of health and nutrition services in Uttar Pradesh, India, despite adaptations to restore services. Strengthening logistical support, capacity enhancement, performance management, and demand creation are needed to improve service provision and utilization during and post-COVID-19.

## Introduction

The coronavirus (COVID-19) pandemic has affected health systems in multiple ways, including reductions in the workforce, supplies, demand, and access (1–3). Mortality and morbidity are exacerbated, both directly from the outbreak and indirectly from other communicable and preventable diseases, due to changes in priority of care (2), isolation, travel restrictions, interruptions in communication among providers or between providers and patients, interruptions in access to medicines and technologies, and economic slowdowns (3). Early estimates suggest that potential disruptions of health systems and essential services could lead to 1,157,000 additional child deaths and 56,700 additional maternal deaths (1). In addition, a 10% decline in the use of sexual and reproductive health care services in 132 low- and-middle income countries (LMICs) would result in annual impacts of 48 million additional women with an unmet need for modern contraceptives, 15 million additional unwanted pregnancies, and >3 million additional unsafe abortions (4). These estimates, however, were based on assumptions because no data on disruptions in service provision existed. The concerning implications of COVID-19 on health services increase multifold in the context of LMICs with weak pre-existing health systems and high burdens of infections (5, 6).

Existing health systems have been overwhelmed by the pandemic, and efforts are being made to ramp up their capacities, especially the government facilities, to both treat COVID-19 patients and maintain essential primary care services (7). At the helm of the basic fabric of health care systems lie the frontline workers (FLWs), tasked with multiple responsibilities of catering to the basic health and nutrition needs of the community (8). FLWs also play a critical role in fighting the pandemic with additional COVID-19 responsibilities such as health surveys, quarantine duties, surveillance, and behavior change communication (3), thus they are particularly vulnerable to the physical, mental, and emotional impacts associated with COVID-19 (9–11). FLWs responding to COVID-19 are reported to be at increased risk of contracting the virus (9, 12) and experience fatigue, uncertainty, attacks, and sometimes conflicts about serving patients or securing their own lives and families (13, 14). Beyond the risk of exposure, COVID-19 also has significant implications for the psychological health of FLWs including increased risk of trauma, stress-related disorders, depression, and anxiety (11, 15).

Whereas several studies have documented physical or psychological impacts of COVID-19 on FLWs (11, 14–16), limited information exists on the impact of COVID-19 on service provision. One such study used a rapid online global survey to qualitatively document changes in service and care processes during COVID-19 as reported by health professionals (5). Those findings, however, mainly came from higher-qualified cadres of health professionals rather than from professionals working in lower-level facilities, particularly in LMICs, owing to limited use of technology or internet access, and barriers caused by the languages used in the survey. A WHO pulse survey across 105 countries reported disruptions in essential health services, particularly in LMICs (17). These findings represented qualitative perspectives of key informants among ministry of health officials only, however, highlighting the need for empirical evidence about the extent of disruption to service provision and utilization at the community level where service provision occurs.

Supply side disruptions, travel restrictions, and reduced mobilization exacerbated risks of inadequate access and utilization of essential health and nutrition services (18), particularly among vulnerable groups including pregnant, lactating women and children (19). On the demand side, households are facing similar disruptions in receipt of services, together with a range of negative impacts on livelihoods, employment, food security, and overall health status (19, 20). Whereas previous studies have provided estimates on the impact of COVID-19 on food insecurity, health care, and health outcomes (20), to our knowledge no study exists on population-level uptake of health and nutrition services.

India is facing double crises in responding to COVID-19 amidst ongoing health systems challenges (21), carrying the second highest burden of COVID-19 in the world with nearly 8 million total confirmed cases and 119,502 deaths as of 16 October, 2020 (22). The Indian government has taken several measures to ensure continued delivery of essential services during the pandemic, but little is known about both provision and utilization of key nutrition intervention services during the pandemic (23). This study aimed to *1*) determine the extent of disruption in provision and utilization of health and nutrition services induced by the pandemic in Uttar Pradesh, a populous state with >200 million people and about one-fifth of India's annual birth cohort; and *2*) identify how adaptations were made to restore health and nutrition service provision in response to the pandemic. The study was part of a larger study in 7 states of FLW service provision (Avula R, Nguyen PH, Ashok S, Bajaj S, Kachwaha S, Pant A, Walia M, Augustine LF, Das S, Krishnan S et al. unpublished results, 2016); Uttar Pradesh was the only state with longitudinal data on both FLWs and mothers of young children.

## Methods

### Study context

Between 2017 and 2019, the Alive & Thrive initiative implemented an intervention to strengthen delivery of maternal nutrition services through the government antenatal care (ANC) platform in Uttar Pradesh, India. In-person baseline and endline surveys were conducted as part of an evaluation to assess the impact of the intervention (12). The endline survey was completed in December 2019, just before the onset of COVID-19. The pre-existing contacts and data availability before the pandemic provided a unique opportunity for a follow-up study to assess the impacts of the COVID-19 pandemic on health and nutrition service delivery by FLWs and on its utilization by mothers of young children.

### Design and data sources

This study used multiple methods, including quantitative surveys with FLWs and mothers of children <2 y old, analysis of data from the Health Management Information System (HMIS—an Indian administrative health data set), and key informant interviews with block-level managers. Data about service provision and adaptation on the supply side came from FLW surveys, covering 3 time points: pre-COVID-19 (December 2019), during the nationwide lockdown (April 2020), and 1 mo before the survey when lockdown norms were relaxed (July 2020). To supplement the findings on service provision, HMIS data (from December 2019 to June 2020, the latest data made available for download in September 2020) were used. Data about service utilization and challenges on the demand side came from surveys of mothers with young infants <2 y old, covering a similar timeline to the FLW surveys. Finally, additional managerial insights on challenges and adaptations to overcome the hardships during the pandemic came from qualitative interviews with key informants. Details of each data source are presented below.

#### FLW surveys

Longitudinal quantitative surveys were conducted with 3 types of FLWs—Anganwadi workers (AWWs), Accredited Social Health Activists, and Auxiliary Nurse Midwives (ANMs)—who are the key service providers for essential health and nutrition services at the grassroots level in India. The study site was in all 26 blocks from the 2 districts (Kanpur-Dehat and Unnao) in Uttar Pradesh. Data were collected through in-person interviews in December 2019 and phone-based interviews in July 2020. Of the 479 FLWs assessed during the in-person survey in December 2019, 320 (65%) of them were also assessed during the phone survey in July 2020. Reasons for nonresponse in the phone survey were contact number unavailable (*n* = 41), phone unreachable or switched off (*n* = 130), wrong number (*n* = 14), no response/request for a reschedule (*n* = 16), refusal to participate (*n* = 3), and respondent died/stopped working as an FLW (*n* = 6) (**Supplemental Figure 1**). The final analytical sample consisted of 313 FLWs who completed an in-person interview in December 2019 and a phone interview in August 2020.

Questionnaires for FLWs were designed in accordance with an overall framework for data collection (Avula R, Nguyen PH, Ashok S, Bajaj S, Kachwaha S, Pant A, Walia M, Augustine LF, Das S, Krishnan S et al. unpublished results, 2016). We collected information on the changes in service provision, challenges, adaptations to health and nutrition service provision, and training and knowledge on COVID-19. We asked FLWs about several activities that they undertook across the continuum of care (from preconception, during pregnancy and delivery, during childhood), via various platforms [through rural child care centers known as Anganwadi Centers, community events like Village Health and Nutrition Days (VHNDs), and home visits]. We also collected information on food supplementation [such as take-home rations (THRs) and hot cooked meals] and social protection.

#### Surveys with mothers of children <2 y old

The survey with mothers of children <2 y old was conducted following the same study design and sampling frame as in the main impact evaluation study (12). Of the 1849 mothers who were interviewed in person in December 2019, 587 mothers were interviewed over the phone in July 2020, yielding a response rate of 32% (Supplemental Figure 1). Reasons for nonresponse were contact number unavailable (*n* = 388), phone unreachable or switched off (*n* = 666), wrong number (*n* = 136), requested a reschedule/no response (*n* = 53), refusal to participate (*n* = 53), and child death (*n* = 9). We also excluded 18 mothers who were pregnant during the phone survey in 2020. The sample of 569 nonpregnant mothers interviewed in both surveys was used for analysis.

We collected information on receipt of services for children <2 y old including health and nutrition services, counseling on child feeding, food supplementation, and social protection. We also collected data on modes of receiving services and challenges faced in accessing services.

#### Administrative health data

HMIS data were downloaded on 28 August, 2020. We focused our analysis on the provision of 6 health services for which data are available in both the HMIS and FLW survey: organizing VHNDs, distribution of family planning products, ANC checkup, provision of iron–folic acid (IFA) supplementation and tetanus immunization during pregnancy, and child immunization.

#### Key informant interviews with block managers

We conducted in-depth telephonic interviews with 6 managers at the block level in the 2 districts to understand challenges and innovations in service delivery during the COVID-19 pandemic. We drew a convenience sample from a sampling frame of 37 block managers from the Integrated Child Development Services and Health Department, including 3 child development protection officers, 2 block program managers, and 1 block community process manager. The interview guideline covered the operational status of services since COVID-19, challenges in service provision and utilization, and additional support required.

### Data analysis

Descriptive analysis was conducted to report characteristics of FLWs and mothers. We compared background characteristics of the analytic sample with those lost to follow-up using Student's *t* test (for continuous variables) and the chi-square test (for categorical variables). We used the Wilcoxon matched-pairs signed rank test to examine changes in service provision and utilization for 3 periods: *1*) between December 2019 and April 2020, to assess service disruption due to the COVID-19 lockdown; *2*) between April and July 2020 (when lockdown norms were relaxed), to assess service resumption; and *3*) between December 2019 and July 2020, to compare the service provision before and after the pandemic. The trends in service provision were also assessed using the HMIS data from December 2019 to June 2020 and compared with the findings from the FLW survey. Statistical analysis was undertaken using Stata version 16 (StataCorp LLC). Significance levels were set at 5%.

The transcripts from the interviews with block managers were compiled in Microsoft Word, and data were summarized along 3 broad themes: adaptation, challenges, and support required to provide services such as organizing community events, home visits, social protection, ANC, and child-related services.

### Ethical clearance

Informed consent in the local language was obtained from mothers, FLWs, and block managers before their participation in the study. The research protocol received ethical clearance from the Institutional Review Board at the International Food Policy Research Institute and the Suraksha Independent Ethics Committee in India. Additional permissions for data collection were provided by the State Government of Uttar Pradesh.

## Results

### Characteristics of the study sample

Approximately 80% of FLWs received secondary- and graduate-level education. FLWs in the analytical sample were slightly younger (40.4 compared with 42.6 y old) with shorter working duration (13 compared with 14.5 y) than in the nonanalytic sample (**Supplemental Table 1**). Mothers were ∼26 y old and their children were ∼3 mo old in December 2019. More than 90% of mothers were housewives. Mothers in the analytic sample had higher education (8.2 compared with 6.7 y of schooling) and lived in wealthier households (51% compared with 35% in quintiles 4–5) than in the nonanalytic sample. FLWs and mothers belonging to the intervention and control areas of the impact evaluation were proportionately represented in the analytic sample.

### Changes in service delivery during the COVID-19 pandemic

Between December 2019 and April 2020, almost all service provisions reduced substantially (**Table 1**). During the lockdown, only 4% of FLWs reported providing VHND services, 29% conducted home visits, 1% ANC services, and 5% child growth monitoring, corresponding to reductions of 93, 69, 99, and 53 percentage points (pp), respectively. The disruption in service provision was similar across all FLWs (**Supplemental Table 2**). In contrast, the provision of THRs was higher in April and July 2020 than in December 2019 (71% and 97% compared with 57%, respectively). All service delivery resumed significantly since July 2020 but was still lower than during the prepandemic period.

**TABLE 1:** Service delivery before the coronavirus pandemic, during the lockdown, and in the previous month, according to the in-person survey in December 2019 and phone survey in August 2020^1^

	FLWs who reported providing this service in:			Changes between:		
	Dec 2019, %	Apr 2020, %	Jul 2020, %	Dec 2019–Apr 2020, pp	Apr 2020–Jul 2020, pp	Dec 2019–Jul 2020, pp
Overall						
Opened Anganwadi Centre, %	100.0	18.0	89.2	−82.0***	71.2***	−10.8***
Conducted VHNDs, %	96.2	3.5	89.1	−92.7***	85.6***	−7.0***
Made home visits, %	98.4	29.1	84.4	−69.3***	55.3***	−14.1***
Counseling on health and nutrition, %	100.0	13.4	96.2	−86.4***	82.8***	−3.8***
Preconception						
Distributed family planning products,^2^ %	46.0	8.4	84.7	−37.6***	76.2***	38.6***
IFA supplementation for adolescents, %	NA	3.5	49.7	NA	46.2***	NA
Pregnant and delivery						
ANC checkups,^3^ %	100.0	1.2	77.7	−98.8***	76.5***	−22.4***
IFA supplementation for pregnant women,^2^ %	96.5	3.5	85.6	−93.1***	82.2***	−10.9***
Immunization services, %	89.5	4.2	93.0	−85.3***	88.8***	3.51
Childhood						
Growth monitoring, %	54.6	4.5	52.7	−50.2***	48.3***	−1.9
Referred malnourished cases, %	10.2	1.5	8.9	−8.7***	7.4***	−1.4
Immunization services, %	88.5	3.8	84.7	−84.7***	80.9***	−3.8***
ORS/ORS and zinc to diarrhea, %	7.0	1.9	31.3	−5.1**	29.4***	24.3***
Social protection^4^						
THRs, %	56.8	71.2	97.3	14.4*	26.1***	40.5***
Hot cooked meal, %	NA	0.0	1.8	NA	1.8	NA
Dry ration,^5^ %	NA	22.6	50.9	NA	28.4***	NA

1 Values are percentages or percentage points. Reported services during the in-person survey in December 2019 were asked using unprompted multiple-choice questions; services during the phone survey in August 2020 were asked using prompted yes/no questions. ^*,**,***^Significantly different: *P < 0.05; **P < 0.01; ***P < 0.001. ANC, antenatal care; ANM, Auxiliary Nurse Midwife; FLW, frontline worker; IFA, iron–folic acid; NA, not applicable; ORS, oral rehydration solution; THR, take-home ration; VHND, Village Health and Nutrition Day.

2 Survey among ANMs and Accredited Social Health Activists (*n* = 202).

3 Survey among ANMs (*n* = 85).

4 Survey among Anganwadi workers (*n* = 111).

5 Dry ration/cash received during lockdown was asked only if hot cooked meal was not received during lockdown.

**FIGURE 1:**
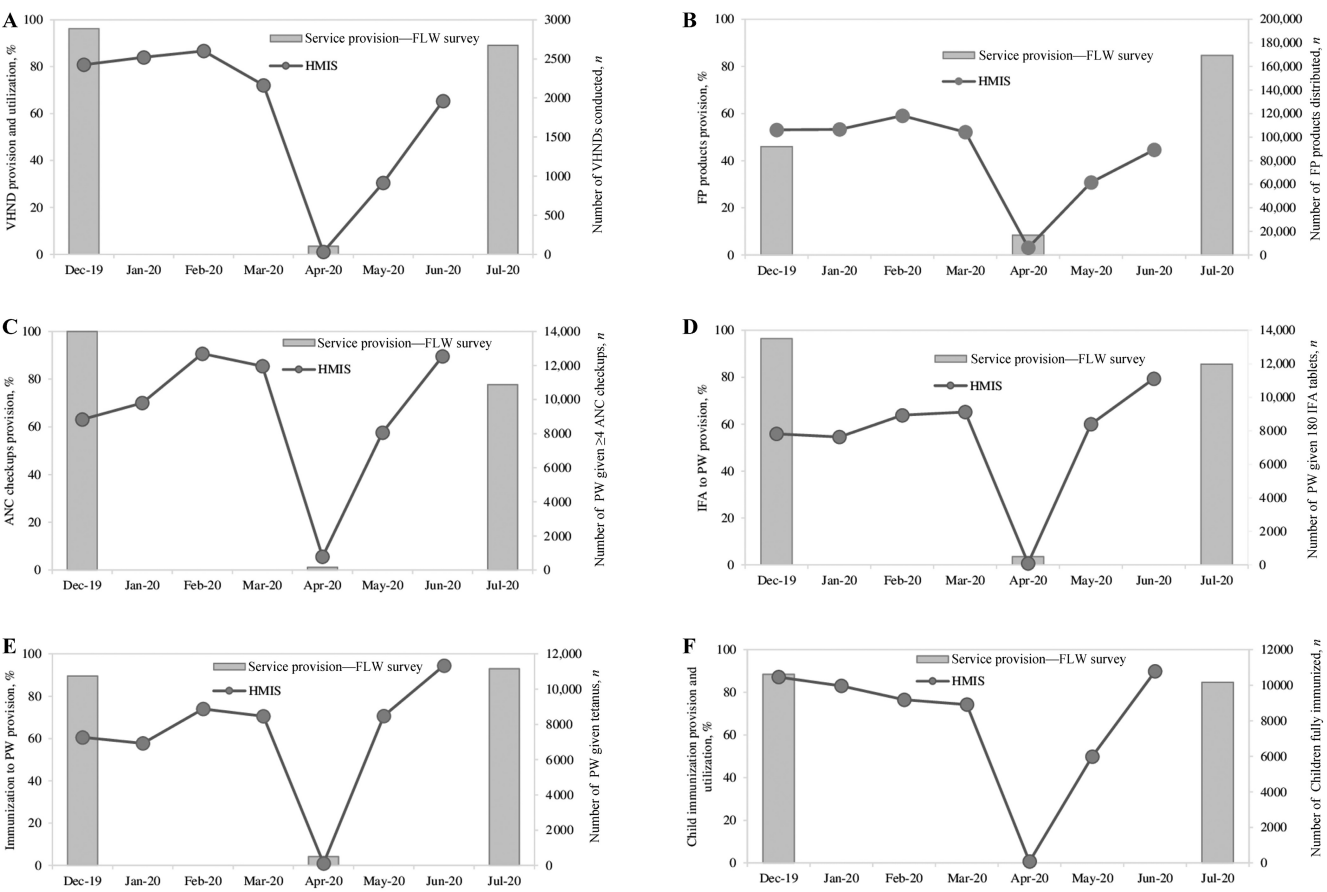
Service delivery before and during the coronavirus pandemic, comparing FLW surveys and HMIS data. (A) Conducted VHNDs, (B) family planning services, (C) ANC checkups for pregnant women, (D) IFA supplementation during pregnancy, (E) tetanus immunization during pregnancy, (F) child immunization. Values are numbers or percentages (*n* = 313). ANC, antenatal care; FLW, frontline worker; FP, family planning; HMIS, Health Management Information System; IFA, iron–folic acid; PW, pregnant women; VHND, Village Health and Nutrition Day.

HMIS data showed a similar pattern of service disruption during the lockdown and gradual resumption over subsequent months (**Figure 1**). For example, the number of VHNDs or ANC checkups conducted fell sharply during April 2020 and picked up gradually in May and June 2020 but was not yet at prepandemic levels. Similar patterns were observed for services such as family planning, ANC checkups, IFA supplementation and tetanus injection during pregnancy, and child immunization.

### Adaptations to restore service provision

**FIGURE 2:**
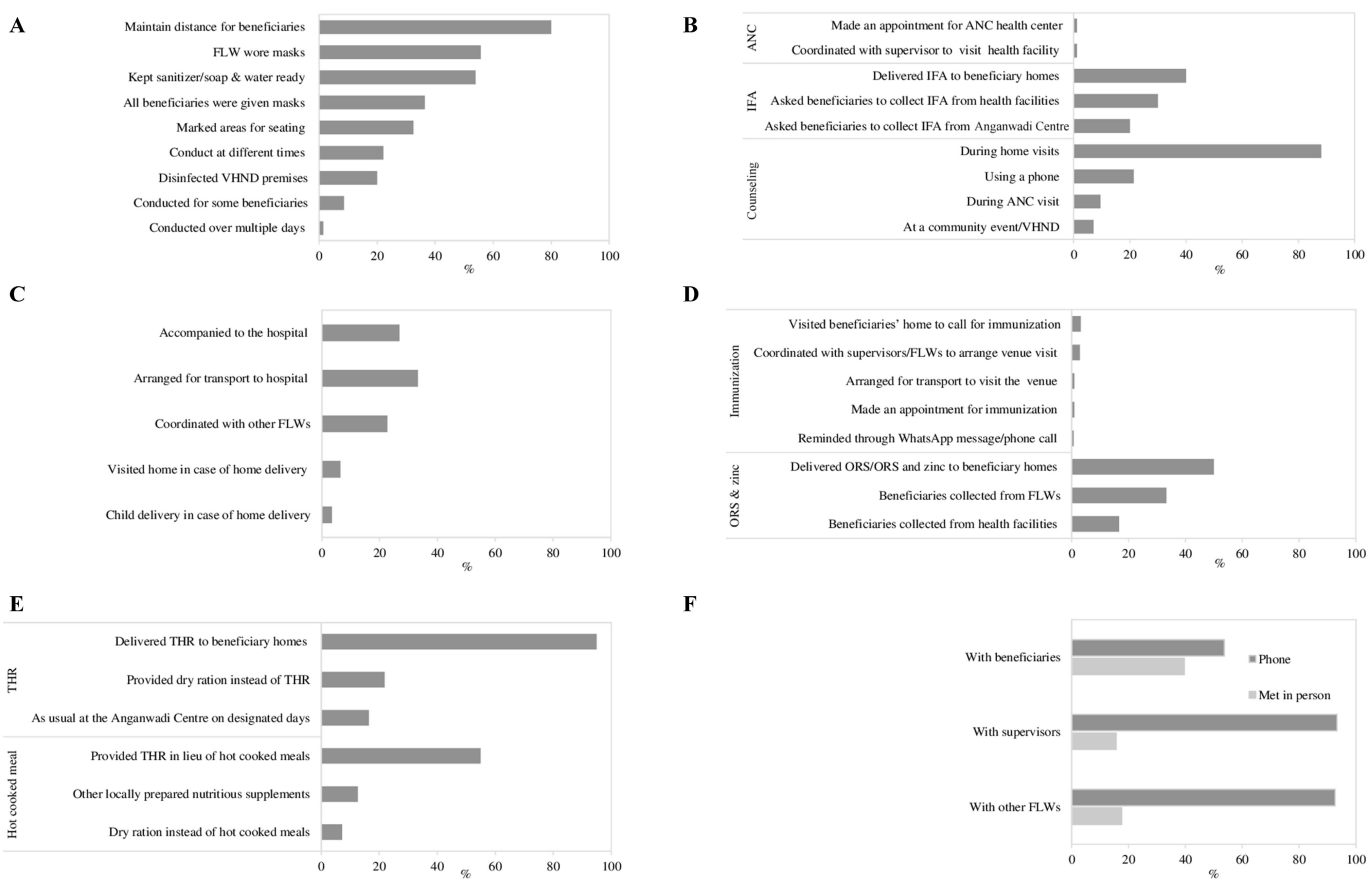
Adaptations made to provide services to beneficiaries during the pandemic. (A) Conducted VHNDs; (B) pregnancy-related services: ANC, counseling, and IFA; (C) delivery; (D) child-related services; (E) food supplementation; (F) means of communication. Values are percentages (*n* = 313). ANC, antenatal care; FLW, frontline health worker; IFA, iron–folic acid; ORS, oral rehydration solution; THR, take-home ration; VHND, Village Health and Nutrition Day.

During the lockdown, FLWs made several adaptations to restore service provision at the community level (**Figure 2**, **Supplemental Table 3**). For VHNDs, 80% of FLWs asked beneficiaries to maintain distance, 56% wore masks, and 54% kept sanitizer/soap and water ready. FLWs also increased outreach activities such as delivery of IFA (40%), oral rehydration solution and zinc (50%), THRs (95%), or counseling services (80%) to beneficiaries’ homes. In addition, FLWs changed the mode of communication with their peers, supervisors, and beneficiaries by using a phone instead of meeting in person.

Discussions with block managers revealed that the key adaptation strategy was prioritization of the most vulnerable beneficiaries (pregnant women in the third trimester, high-risk pregnancies, malnourished children), as well as of those who were due for services (**Table 2**). This applied to provision of both facility and outreach services:

**TABLE 2:** Insights from qualitative interviews with block-level managers on service delivery during the COVID-19 pandemic^1^

Services	Adaptation for service delivery	Challenges in service delivery	Additional supports needed for service delivery
VHND services	• Prioritized beneficiaries who were not yet due to receive services• FLWs built trust among the beneficiaries and motivated them• Staff members kept soap and water buckets ready• FLWs wore masks and gloves and sanitized their hands when providing services• In the areas where participation was low, UNICEF and WHO teams motivated beneficiaries	• Less cooperation from beneficiaries—resulted in less coverage and poor data collection• Scared to participate, especially families with infants• Beneficiaries didn't wear masks• Fear of infection among FLWs• Lack of PPE for FLWs• Lack of incentive for FLWs	• Provide PPE and incentives to FLWs• Senior block-level officers should participate more often in VHNDs and motivate beneficiaries• Reduce workload of FLWs• Designate a place in the village for the VHND—equipment should be available there• Support of NGOs/other organizations in the provision of counseling during VHNDs
Home visits	• Prioritized high-risk beneficiaries such as pregnant women and malnourished children• No routine home visits took place. FLWs visited houses to conduct the COVID-19 survey and during that distributed IFA/calcium tablets	• Less cooperation from beneficiaries—FLWs were told to return, beneficiaries used abusive language• Lack of transport• Increased workload among FLWs (distribute THRs, collect data, and maintain records of beneficiaries’ contact numbers for validation)—unable to cover all beneficiaries	• Provide PPE to FLWs and some masks for beneficiaries• Reduce interference from other work or calls from supervisor during home visits by FLWs—this will help FLWs carry out their duties effectively
THRs and hot cooked meal	• No disruption in THR provision since the pandemic—3 packets/mo provided• THRs delivered at home• Hot cooked meals not being provided	• No transport to deliver THRs at beneficiaries’ homes• Lack of budget (not able to provide hot cooked meals)• Increased workload among FLWs (distribute THRs, collect data, and maintain records of beneficiaries’ contact numbers for validation)	• Provide THRs at Anganwadi Centres—door-to-door delivery may increase the risk of contracting the virus• Improve the quality of THRs• Bridge ration supply gaps• Provide regular budget for hot cooked meals
ANC	• Prioritized high-risk beneficiaries, e.g., women in the third trimester• Frequent follow-ups, counseling (COVID-19 precautions, diet, adequate rest, IFA intake), and addressing of urgent needs (connect with a gynecologist, arrange ambulance) over phone• ASHAs gave diet-related posters to pregnant women	• Less cooperation from beneficiaries (scared, not ready for a health checkup)• Manpower shortage—FLWs and doctors in the facility• Outpatient department was closed on a few days—could not give all ANC services	• Senior block officers should motivate beneficiaries to take up ANC service• Fill ANM vacancies
Child health services	• Prioritized children due for immunization since April• Under the government campaigns, FLWs did line listing of pending immunizations (20 houses in 1 go). In areas where the dropout rate was high, services were provided• Persuaded beneficiaries to participate—explained the importance of immunization, gave a fake threat of canceling the public distribution system ration	• Limited transport facility—disrupted immunization service, child referral to Nutrition Rehabilitation Centre• Low vaccine supply• Shortage of manpower• Less cooperation from beneficiaries (fearful about catching the virus)• Lower attendance of children in VHNDs—pressure to conduct growth monitoring at home (not feasible because of unleveled floors)	• Fill ANM vacancies• Give incentive to FLWs• Provide PPE to FLWs—will help to gain beneficiaries’ cooperation

1 *n* = 6: 3 child development protection officers, 2 block program managers, and 1 block community process manager. ANC, antenatal care; ANM, Auxiliary Nurse Midwife; ASHA, Accredited Social Health Activist; COVID-19, coronavirus; FLW, frontline worker; IFA, iron–folic acid; NGO, nongovernmental organization; PPE, personal protective equipment; THR, take-home ration; VHND, Village Health and Nutrition Day.

*Under government COVID-19 campaigns, FLWs have been doing line listing of children 0–5 y, missed ANCs, and pending immunizations. We do gap-analysis of the pending immunizations. And based on the listing, in the areas where needed (high dropout rate), immunization/other service is provided via VHND/subcenters by ANMs*. (Block community process manager)

Beneficiaries who were scared to participate were motivated by FLWs and other development organizations. Because VHNDs were held in a community setup, following COVID-19 precautions such as wearing masks and keeping soap and water buckets handy helped FLWs in building trust among beneficiaries. In areas where routine home visits did not take place, IFA and calcium tablets were provided during the visit for the COVID-19 survey. FLWs said they contacted pregnant women frequently via phone and counseled them on COVID-19 precautions, diet, rest, and IFA intake. THRs were provided at home, ensuring smooth provision even during the lockdown:

*Staff members keep soap and water bucket handy* [during VHNDs]. *Only if they have precautionary measures, registers are provided to them. FLWs motivated beneficiaries to wear masks*. (Child development protection officer)

*Beneficiaries didn't object to take THR because of 2 reasons: (1) it was packed, (2) there was shortage of food*. (Child development protection officer)

### Changes in service utilization during the COVID-19 pandemic

**FIGURE 3:**
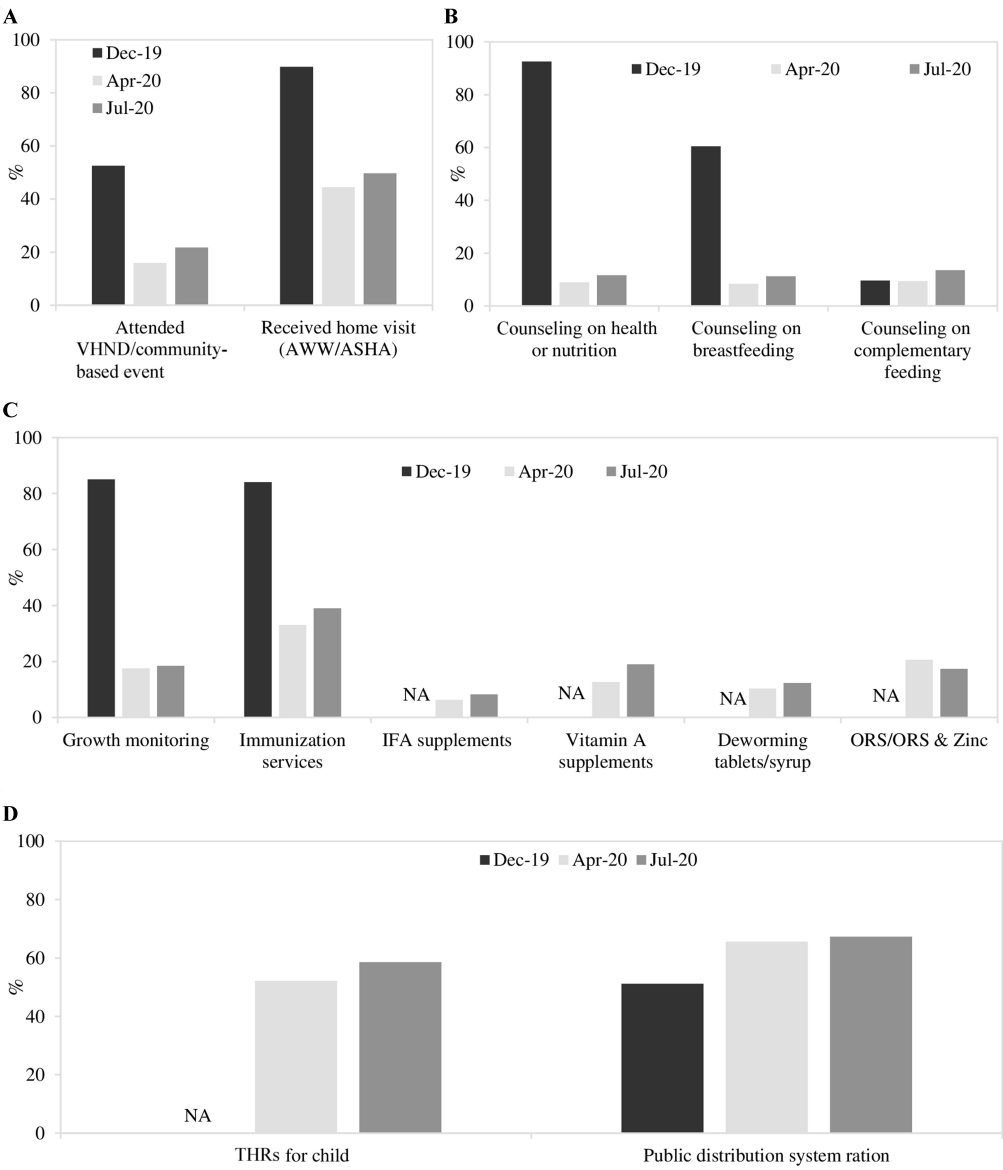
Services received by mothers before the coronavirus pandemic, during the lockdown, and in the previous month, according to the in-person survey in December 2019 and phone survey in August 2020. (A) Overall, (B) counseling, (C) child health and nutrition, (D) food supplementations and social protection services. Values are percentages (*n* = 569). ASHA, Accredited Social Health Activist; AWW, Anganwadi worker; IFA, iron–folic acid; NA, not applicable; ORS, oral rehydration solution; THR, take-home ration; VHND, Village Health and Nutrition Day.

Service utilization for most services dropped considerably during the pandemic lockdown (84 pp reduction in counseling on health or nutrition, 67 pp in growth monitoring, 51 pp in child immunization, 45 pp in receiving home visits, and 37 pp in attending VHNDs) (**Figure 3**, **Supplemental Table 4**). In contrast, more mothers received food rations in July 2020 than prepandemic (66% compared with 51%). More than half of mothers received THRs for their children during the lockdown. Utilization for most services improved slightly between lockdown and July 2020 (∼5 pp).

### Challenges in service provision and utilization related to the COVID-19 pandemic

**FIGURE 4:**
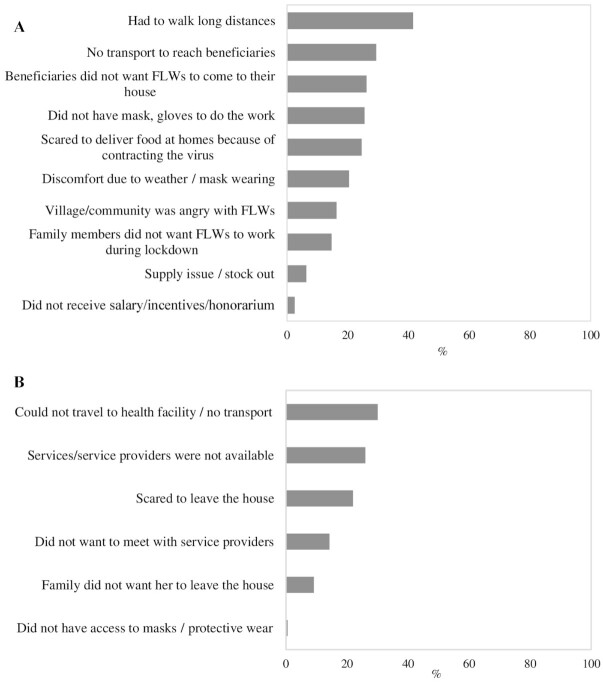
Challenges faced in service provision and utilization during the pandemic lockdown. Challenges faced by (A) FLWs and (B) mothers. Values are percentages. FLW, frontline worker.

On the supply side, challenges faced by FLWs in service provision were having to walk long distances (42%), nonavailability of transportation (29%), less cooperation from beneficiaries (26%), lack of personal protective equipment (PPE) (26%), fear of contracting the virus while delivering food to beneficiaries (25%), and discomfort wearing a mask (20%) (**Figure 4**).

On the demand side, challenges faced by beneficiaries in utilizing services were difficulty in travelling to a facility (30%), nonavailability of services or providers (26%), fear of leaving the house (22%), and reluctance to meet service providers (14%).

Block managers in interviews reported that service provision since COVID-19 was disrupted mainly owing to less cooperation from beneficiaries, because beneficiaries were fearful of catching the virus, especially families with infants (Table 2):

*Beneficiaries told AWWs to go back and threw away their registers. Some of them even used abusive language*. (Child development protection officer)

In addition, managers reported increased FLW workload and manpower shortages among the challenges in service provision during the pandemic:

*Workload has increased, now we have additional duties of line listing and record maintenance. Could not cover all beneficiaries due to manpower shortage*. (Block community process manager)

No transport arrangements were available for delivery of THRs at beneficiaries’ homes, thus FLWs had to carry heavy food packages on their own. Limited transport facilities also disrupted immunization provision and child referral. Lack of PPE hindered implementation of VHNDs because FLWs were afraid of catching the infection.

### Resources and FLW knowledge to respond to COVID-19

Most FLWs (>80%) received training on COVID-19 symptoms and protection (**Supplemental Figure 2**). FLWs had good knowledge on common methods of protection against COVID-19: cleaning hands with sanitizer (85%), maintaining physical distance (82%), wearing a mask (79%), and washing hands with soap (71%). Whereas FLWs were well trained on COVID-19, they were poorly equipped to provide services: only 60% of FLWs received face masks, 38% received sanitizer/soap, 35% received gloves, and 3% had face shields.

### Additional support required for service provision

**FIGURE 5:**
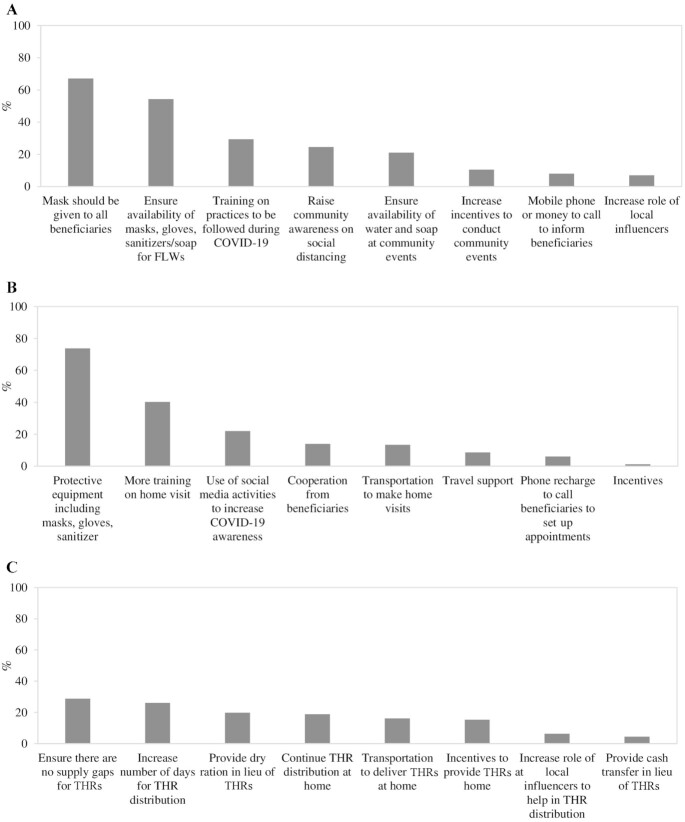
Additional resources or support needed by FLWs to provide services. (A) Organizing VHNDs, (B) home visits, and (C) THR distribution. Values are percentages (*n* = 313). COVID-19, coronavirus; FLW, frontline worker; THR, take-home ration; VHND, Village Health and Nutrition Day.

The additional support FLWs needed to provide services during the pandemic included adequate PPE for FLWs (54%) and beneficiaries (67%) (**Figure 5**). Other support needed included provision of more training on organizing VHNDs (29%) or home visits (40%), raising community awareness, and ensuring adequate supplies. Some FLWs also expressed they needed support for transportation to make home visits (∼15%) and travel support (∼10%).

Block manager interviews highlighted areas of special support needed, including the urgent need for PPE for FLWs and beneficiaries, considering additional incentives for FLWs, involvement of senior block officers in community events, motivating beneficiaries to participate, and support from other organizations. Block managers also expressed concerns about THR quality and supply gaps and called for support to improve THR quality, improve supply, and provide budget for hot cooked meals. In addition, managers reported filling FLW vacancies with urgency as among the critical support needed for service delivery during the pandemic (Table 2).

## Discussion

This study provides unique and timely insights on provision and utilization of essential health and nutrition services before and during the COVID-19 pandemic, covering the prepandemic, lockdown, and postlockdown periods. We observed significant disruptions in service provision during the lockdown for facility-based services, but outreach-based services continued. Service provision for most services resumed in July 2020, except for immunization and hot cooked meals, which continue to lag. Administrative data showed similar patterns of service disruption and gradual resumption over this period. Several adaptations were used for service provision including home delivery, ensuring social distancing, using PPE for both FLWs and beneficiaries, and using phones. Our study provides important insights on the demand side, showing that household-level service utilization also reduced substantially during the lockdown. In contrast with insights on restored service provision, household coverage of most services had not improved significantly even 4 mo after the lockdown.

Facility-based services were substantially affected during the lockdown, mainly due to suspension of services in areas with positive COVID-19 cases by the state government (23). In contrast with findings from other studies suggesting lack of training limited health workers’ ability to perform duties during COVID-19 (13, 24), most FLWs in our study received training and had good knowledge of COVID-19. Receipt of PPE and incentives were inadequate, however, which hindered effective service provision, which is consistent with findings from other studies in India (25) and Pakistan (13). Other factors contributing to service reduction included shortages of manpower and supply, increased workload among FLWs, lack of transport, and poor cooperation from beneficiaries. Our findings highlight the need of particular attention to address the reported challenges expressed by FLWs and of urgency to fill vacancies to maximize providers’ ability to respond to emergency situations and ensure the provision of essential services.

Outreach-based services remained at different levels for various services. Findings from another state, Chhattisgarh, also showed a drop in subcenter immunization in early April but outreach sessions were less affected (26). Provision of THRs in particular was high during lockdown and actually increased from before to during the COVID-19 time, which was explained by national and state government efforts to initiate home delivery of THRs during the pandemic (23). The provision of oral rehydration solution was significantly higher in July 2020 than in December 2019, corresponding to the increase in diarrhea cases (∼280%) as shown in the HMIS data.

Several innovative adaptations were implemented in accordance with state policy guidelines to deliver services during the pandemic. The Uttar Pradesh state policy directives (released in April 2020) emphasize the focus of essential health care services as targeting vulnerable beneficiaries (high-risk pregnant women, newborns, and young children) during the lockdown, and encourage the continuation of other services (community-based ANC checkups, home-based newborn care, immunization, and VHNDs) with precaution in areas with no COVID-19 cases (23). Given the restrictions on movement and contacting people, the mode of communication changed from in-person to phone. A systematic review of the potential benefits of mobile health initiatives in India found many apps that have been developed to prevent and manage the COVID-19 pandemic (27). Comprehensive mHealth solutions for FLWs could be applied for health and nutrition services as well.

For service utilization, we observed a considerable drop without much improvement after the lockdown. The key reasons for disruption of service utilization included fear of infection among beneficiaries, resistance to meet FLWs, and unavailability of services/providers. The only service with increased utilization during the pandemic was food rations, which points to the importance of social protection programs during the pandemic. A recent study conducted in the same sample found household food insecurity increased sharply by 60% during the pandemic and households engaged in several coping strategies to obtain food (28). These findings potentially explain why uptake of food rations increased in this period whereas use of other preventative services was not similarly prioritized by households. Our results indicate the importance of demand creation in restoring service utilization while bearing the impacts of COVID-19. Generating awareness in the community to assure beneficiaries of precautionary measures through mass-media and engagement of local village leaders may help generate demand and improve utilization. Effective coordination and a comprehensive infection plan are imperative during a pandemic (29) to protect pregnant women, mothers, and children in LMICs who may have higher nutrition-related risk factors for poor outcomes related to COVID-19 (30).

The major disruptions observed in health and nutrition services during COVID-19 have important implications for the nutritional status of mothers and children in India. Based on a previous modeling study (1), the service disruption in India was severe (40%–80%); severe service disruption would result in ∼50,000 additional child deaths and ∼2400 additional maternal deaths in 1 mo, accounting for one-quarter of the estimated deaths across 118 LMICs. Disruptions in maternal and child health services may also result in higher morbidity and mortality from other diseases, particularly among vulnerable groups including young children and pregnant women who are the most in need of health care (31). In addition to negative impacts on mortality, persistent disruptions in health and nutrition services could lead to adverse fetal outcomes including preterm birth, low birth weight, and small-for-gestational-age newborns (18). Finally, the interlinkages between health care systems and food insecurity may further exacerbate existing social and health inequities (19).

The mixed methods, longitudinal design, and multiple data sources used in our study make it uniquely able to provide insights on the extent of disruptions and utilization of services during the pandemic, offering one of the first empirical investigations on this critical topic. Triangulation across the mixed methods, longitudinal design, and multiple data sources provides justification for plausibly inferring that the disruptions to service provision and utilization were attributable to the pandemic and that the adaptations made restored them. Using a longitudinal sample of FLWs, we have shown a trend of service provision during 3 critical time periods: before COVID-19 in December 2019, during the lockdown in April 2020, and after lockdown in July 2020. This trend estimated from FLW survey data was corroborated by longitudinal administrative health data. No events in Uttar Pradesh other than the pandemic occurred during the study period that could alternatively explain the findings. Although both in-person and telephonic surveys were used, the questionnaires used asked similar questions. Results from the phone survey were comparable with those from the administrative data, which provides confidence on the credibility of our findings. Switching from in-person to the telephonic mode of administration has been common during the pandemic and has been undertaken successfully by leading global survey systems including the Gallup World Poll (32). Mode of administration in general has little effect on responses to questionnaire items (33–35). Insights from surveys with FLWs helped to identify innovative adaptations amidst the unprecedented pandemic and also gaps that existed in the provision and utilization of services. Furthermore, the quantitative data were complemented by in-depth interviews with block managers, which highlighted key insights for decision makers and practitioners to understand the experiences of FLWs and beneficiaries and how best to respond to the ongoing challenges during the pandemic. Our study also provides evidence for potential modeling of the impacts on maternal and child undernutrition during the pandemic and to specifically advise policy decisions in India and other developing countries.

During COVID-19, assessment of service provision and utilization was conducted using telephonic surveys. All possible measures were undertaken to get a maximum response rate and to ensure that the quality of the telephonic surveys was comparable with that of the pre-COVID-19 in-person survey. Bearing similar challenges as other phone surveys (36), the response rate of the household survey was low. Comparing background characteristics of respondents interviewed through in-person as opposed to telephonic surveys showed lower education and poorer socioeconomic background in the former group, indicating the difficulty of reaching the poorest or most vulnerable households through telephonic surveys. Furthermore, our findings on low service utilization among respondents reached by the telephonic survey could underestimate the impact on the most vulnerable.

In conclusion, COVID-19 disrupted the provision and utilization of health and nutrition services despite positive adaptations. Service provision restored significantly in July 2020 compared with April 2020 but was still lower than prepandemic provision in December 2019. The utilization of most services had not improved significantly even 4 mo after the lockdown ended, suggesting that major investments are needed to bring beneficiary populations back to use preventive health and nutrition services. Further investments are needed in continuous training, provision of protective equipment and support for delivering services with precautions, performance management for facilities, and outreach services to support service utilization during and post-COVID-19. Creating greater demand for service utilization will require additional research to fully understand and mitigate the demand-side challenges.

## Supplementary Material

nxab135_Supplemental_File

## Notes

Supported by the Bill & Melinda Gates Foundation through Partnerships and Opportunities to Strengthen and Harmonize Actions for Nutrition in India (POSHAN), led by the International Food Policy Research Institute (to PHN). The funders had no role in study design, data collection and analysis, data interpretation, the decision to publish, or preparation of the manuscript.

Author disclosures: the authors report no conflicts of interest.

PHN is a member of the Journal's Editorial Board.

Supplemental Figures 1–2 and Supplemental Tables 1–4 are available from the “Supplementary data” link in the online posting of the article and from the same link in the online table of contents at https://academic.oup.com/jn/.

Abbreviations used: ANC, antenatal care; ANM, Auxiliary Nurse Midwife; AWW, Anganwadi worker; COVID-19, coronavirus; FLW, frontline worker; HMIS, Health Management Information System; IFA, iron and folic acid; LMIC, low- and-middle income country; pp, percentage points; PPE, personal protective equipment; THR, take-home ration; VHND, Village Health and Nutrition Day.
