# Supplementary material for: COVID-19 Disrupted Provision and Utilization of Health and Nutrition Services in Uttar Pradesh, India: Insights from Service Providers, Household Phone Surveys, and Administrative Data
Source: J Nutr. 2021 Jun 3;151(8):2305–16. doi: 10.1093/jn/nxab135 (PMC8195077; doi:10.1093/jn/nxab135)

Supplemental Figure 1: Participant flow for frontline workers (A) and mothers of children <2y (B)

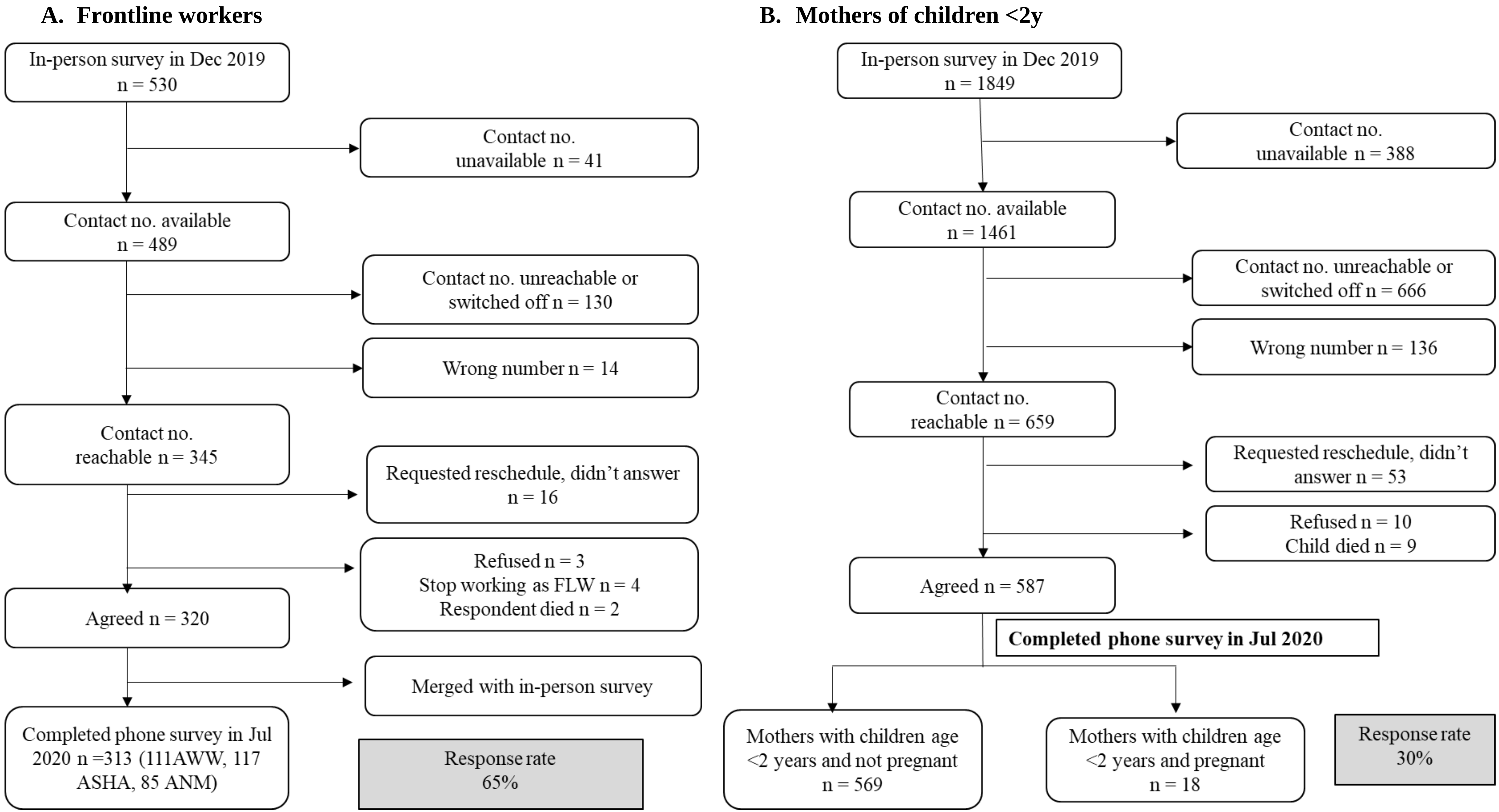

AWW: Anganwadi worker, ASHA: Accredited Social Health Activist, ANM: Auxiliary Nurse Midwife, FLW: Frontline worker

Supplemental Table 1: Background characteristics[1] of the study sample participated in surveys before and during the COVID pandemic (December 2019 and August 2020)[1]

	Analytic sample (both in person and phone surveys before and during the pandemic)	Non-analytic sample (only in person survey before the pandemic)	*p*
**Frontline workers**	***n*= 313**	***n*= 217**	
Age, *y*	40.4 ± 8.8	42.6 ± 9.7	0.009
Highest class completed, %			
Lower than primary	0.64	0.5	0.95
Lower than secondary	19.6	20.8	
Secondary school	40.8	42.1	
Graduate and above	38.9	36.6	
Religion as Hindu, %	97.1	90.8	0.002
Caste category, %			
Scheduled caste/tribe/Other Backward Class	73.3	70.0	0.62
General	26.7	30.0	
Working duration, *y*	12.9 ± 7.9	14.5 ± 9.2	0.041
Maternal nutrition (2017-2019), *n*			
Intervention area	145	122	
Comparison area	168	95	
**Mothers of children <2y**	***n*= 569**	***n*= 1,280**	
Age of respondent mother, *y*	25.5 ± 3.8	25.7 ± 4.0	0.47
Education, *y*	8.2 ± 4.3	6.7 ± 4.6	<0.001
Never attended school, %	14.1	24.8	<0.001
Primary school (grade1-5), %	13.9	16.3	
Middle school (grade 6-9), %	24.3	24.7	
High school (grade 10-12), %	30.1	23.3	
Graduate and above, %	17.8	10.9	
Occupation as housewife, %	91.7	93.0	0.35
Child age, *m*	3.0 ± 1.6	2.8 ± 1.6	0.041
Child sex (male), %	49.0	49.5	0.84
Number of children <5y, *n*	1.6 ± 0.7	1.7 ± 0.7	0.60
Religion as Hindu, %	93.7	91.1	0.061
Caste category, %			
Scheduled caste/tribe	38.3	38.4	0.25
Other Backward Class	44.3	47.0	
General/others	17.4	14.5	
Household socio-economic status, %			
Quintile 1	11.6	23.8	<0.001
Quintile 2	19.2	20.4	
Quintile 3	18.1	20.9	
Quintile 4	24.6	18.0	
Quintile 5	26.5	17.0	
Maternal nutrition (2017-2019), *n*			
Intervention area	282	640	
Comparison area	287	640	

[1]Background data presented in this table were from in -person survey in December 2019, Values are means ± SDs or percentages

Supplemental Table 2: Service delivery, by frontline workers[1]

Indicators	AWW (*n*=111)			ASHA (*n*=117)			ANM (*n*=85)		
	Dec-19	Apr-20	Aug-20	Dec-19	Apr-20	Aug-20	Dec-19	Apr-20	Aug-20
**Overall**									
Opened Anganwadi Centre	NA	18.0	89.2	NA	NA	NA	NA	NA	NA
Conducted VHND	96.4	9.0	87.4	93.2	0.0	85.5	100.0	1.2	96.5
Made home visits	100.0	32.4	81.1	100.0	37.6	89.7	94.1	12.9	81.2
Counselling on health and nutrition	100.0	13.5	92.8	100.0	17.9	98.3	100.0	7.1	97.6
ANC for all women	NA	NA	NA	NA	NA	NA	100.0	1.2	77.6
**Preconception**									
Distributed family planning products	NA	NA	NA	42.7	12.8	82.9	50.6	2.4	87.1
IFA supplementation for adolescent	NA	7.3	57.3	NA	2.6	52.1	NA	0.0	36.5
**Pregnant and lactating mothers**									
ANC for 3rd trimester or high-risk pregnancy	NA	NA	NA	NA	NA	NA	NA	0.0	14.1
Immunization services	89.2	9.0	91.0	82.9	0.0	91.5	98.8	3.5	97.6
IFA supplementation for pregnant women	NA	NA	NA	95.7	4.3	83.8	97.6	2.4	88.2
IFA supplementation for lactating women	NA	8.1	50.5	NA	2.6	67.5	NA	3.5	70.6
Helped women during delivery during lockdown	23.4	5.4	NA	46.2	60.7	NA	24.7	18.8	NA
**Children's health and nutrition**									
Growth monitoring[2]	55.0	11.7	51.4	65.0	0.9	42.7	40.0	0.0	68.2
Referred malnourished cases	10.8	0.0	5.3	8.5	3.2	11.3	11.8	1.6	11.1
Immunization services	89.2	9.0	82.9	82.9	0.0	82.9	95.3	3.5	89.4
IFA supplementation for children	NA	6.3	39.6	NA	0.9	44.4	--	1.2	62.4
ORS/ORS and Zinc to diarrhea	4.5	0.0	16.2	5.1	3.4	28.2	12.9	2.4	55.3
**Social protection**									
Provided THR	56.8	71.2	97.3	NA	NA	NA	NA	NA	NA
Provided hot cooked meal	NA	0.0	1.8	NA	NA	NA	NA	NA	NA
**Village level services for school children**									
Hot cooked meal	NA	1.8	0.0	NA	NA	NA	NA	NA	NA
Dry ration	NA	22.6	50.9	NA	NA	NA	NA	NA	NA
Cash	NA	4.3	10.2	NA	NA	NA	NA	NA	NA

[1] Values are percentages; [2]ASHAs role in growth monitoring is to measure weight of children <42 days only, other measurements are done by AWWs.
VHND: Village health and nutrition day, AWW: Anganwadi worker, ASHA: Accredited Social Health Activist, ANM: Auxiliary Nurse Midwife, ANC: Antenatal care, IFA: Iron-folic acid, NA: Not applicable, ORS: Oral rehydration solution, THR: Take-home rations.

Nguyen et al. Impacts of COVID-19 on provision and utilization of health and nutrition services in Uttar Pradesh, India: Insights from service providers, household phone surveys and administrative data
Online supporting materials

Supplemental Table 3: Adaptations, by frontline workers[1]

	AWW *n*=111	ASHA *n*=117	ANM *n*=85
**VHND**			
VHND was conducted for some beneficiaries only	14.3	4.0	7.3
VHND was organized for different groups of beneficiaries at different times of the day	24.5	16.0	26.8
VHND session was conducted over multiple days to cover the beneficiaries	3.1	1.0	0.0
All beneficiaries were given masks	33.7	33.0	43.9
Beneficiaries were asked to maintain distance	68.4	85.0	87.8
Marked areas for seating	37.8	27.0	32.9
AWW/ASHA/ANM wore masks	53.1	53.0	62.2
Kept sanitizer/soap and water ready	35.7	59.0	69.5
Disinfected the VHND premises	13.3	14.0	35.4
**Counseling**			
During home visits	86.7	95.2	66.7
During ANC visit	0.0	19.0	0.0
At a community event/VHND	20.0	0.0	0.0
Using a phone	20.0	19.0	33.3
**ANC**			
Made an appointment for ANC at the health center	NA	NA	1.2
Arranged for transport to visit the health facility	NA	NA	0.0
Coordinated with ASHA and ANM to arrange for the visit at the health centre	NA	NA	0.0
Coordinated with supervisor to arrange for the visit to the health facility	NA	NA	1.2
Reminded through WhatsApp message/phone call	NA	NA	0.0
Visited beneficiaries' home to call for ANC	NA	NA	0.0
**IFA**			
Delivered IFA to beneficiary homes	NA	50.0	25.0
Beneficiaries were asked to collect it from Anganwadi Centre	NA	16.7	25.0
Beneficiaries were asked to collect it from health facilities	NA	50.0	0.0
Other FLW/ community volunteer delivered it to beneficiary homes	NA	0.0	25.0
**Delivery**			
Accompanied to the hospital	2.7	56.4	17.6
Arranged for transport to the hospital	16.2	51.3	30.6
Coordinated with AWW, ASHA or ANM to accompany them to the hospital	26.1	11.1	34.1
Visited home in case of home delivery	6.3	8.5	3.5
Facilitated child delivery in case of home delivery	2.7	4.3	3.5
**Immunization**			
Reminded through WhatsApp message/phone call	1.8	0.0	0.0
Visited beneficiaries' home to call for immunization	9.0	0.0	0.0
Made an appointment for immunization	1.8	0.0	1.2
Arranged for transport to visit the immunization venue	1.8	0.0	1.2
Coordinated with AWW/ASHA/ANM to arrange for the visit at the immunization venue	7.2	0.0	1.2
Coordinated with supervisor to arrange for the visit at the immunization venue	3.6	0.0	0.0
**ORS and Zinc**			
Delivered ORS/ORS and zinc to beneficiary homes	NA	25.0	100.0
Beneficiaries collected ORS/ORS and zinc from Anganwadi Centre	NA	0.0	50.0
Beneficiaries collected ORS/ORS and zinc from my house	NA	50.0	0.0
Beneficiaries collected ORS/ORS and zinc from health facilities	NA	25.0	0.0

Other FLW or community volunteer delivered ORS/ORS and zinc to beneficiary homes	NA	25.0	0.0
I told beneficiaries how to prepare ORS at home	NA	0.0	0.0
**THR**			
As usual at Anganwadi Centre to provide THR in April COVID-19 lockdown	16.5	NA	NA
Delivered THR to beneficiary homes in April COVID-19 lockdown	94.9	NA	NA
Provided dry ration instead of THR in April COVID-19 lockdown	21.9	NA	NA
**Hot cooked meal**			
Provided THR instead of HCM in April COVID-19 lockdown	55.0	NA	NA
Provided dry ration instead of HCM in April COVID-19 lockdown	7.2	NA	NA
Provided other local nutritious supplement instead of HCM in April COVID-19 lockdown	12.6	NA	NA

[1] Values are percentages; VHND: Village health and nutrition day, AWW: Anganwadi worker, ASHA: Accredited Social Health Activist, ANM: Auxiliary Nurse Midwife, ANC: Antenatal care, FLW: Frontline worker, IFA: Iron-folic acid, NA: Not applicable, ORS: Oral rehydration solution, THR: Take-home rations, HCM: Hot cooked meal.

Supplemental Table 4: Services received by mothers before the COVID pandemic, during the lockdown and in the previous month, in-person survey December 2019 and phone survey August 2020[1]

	% mothers who reported receiving this service in:			Changes between:					
				Dec 2019- Apr 2020		Apr 2020 - Jul 2020		Dec 2019- Jul 2020	
	Dec-19 (%)	Apr-20 (%)	Aug-20 (%)	pp	p-values	pp	p-values	pp	p-values
**Overall**									
Attended VHND/Community based event	52.6	16.0	21.8	-36.6	< 0.001	5.80	0.002	-30.8	< 0.001
Received home visit (AWW/ASHA)	89.8	44.5	49.7	-45.4	< 0.001	5.28	0.022	-40.1	< 0.001
**Child health & nutrition**									
Growth monitoring	85.1	17.6	18.5	-67.5	< 0.001	0.88	0.714	-66.6	< 0.001
Immunization services	84.1	33.0	39.0	-51.1	< 0.001	5.98	0.037	-45.1	< 0.001
IFA supplements	NA	6.33	8.26	NA	NA	1.93	0.135	NA	NA
Vitamin A supplements	NA	12.7	19.0	NA	NA	6.33	0.001	NA	NA
Deworming tablets/syrup	NA	10.4	12.3	NA	NA	1.93	0.305	NA	NA
ORS/ORS & Zinc	NA	20.6	17.4	NA	NA	-3.20	< 0.001	NA	NA
**Counselling for child**									
Counselling on health or nutrition	92.6	8.96	11.6	-83.7	< 0.001	2.64	0.067	-81.0	< 0.001
Counselling on breastfeeding	60.5	8.44	11.3	-52.0	< 0.001	2.81	0.037	-49.2	< 0.001
Counselling on complementary feeding	9.67	9.49	13.5	-0.18	1.000	4.04	0.002	3.86	0.047
**Food supplementations**									
THR for child	NA	52.2	58.6	NA	NA	6.39	0.008	NA	NA
Dry ration in lieu of THR/HCM	NA	2.81	3.16	NA	NA	0.35	0.804	NA	NA
**Social protection services**									
Cash in lieu of THR/HCM	NA	0.35	0.53	NA	NA	0.18	1.000	NA	NA
PDS ration	51.1	65.6	67.3	14.4	< 0.001	1.76	0.143	16.2	< 0.001
Cash transfer from government	35.5	32.0	3.16	-3.51	0.229	-28.8	< 0.001	-32.3	< 0.001
Assistance from NGO	NA	0.53	0.53	NA	NA	0.00	1.000	NA	NA

[1] Values are percentages, *n*=569. VHND: Village health and nutrition day, AWW: Anganwadi worker, ASHA: Accredited Social Health Activist, IFA: Iron-folic acid, NA: Not applicable, ORS: Oral rehydration solution, THR: Take-home rations, HCM: Hot cooked meal, PDS: Public distribution system, NGO: Non-government organization

Nguyen et al. Impacts of COVID-19 on provision and utilization of health and nutrition services in Uttar Pradesh, India: Insights from service providers, household phone surveys and administrative data
Online supporting materials

Supplemental Figure 2: Resources for and knowledge of frontline workers to respond to COVID-19 (*n*=313)

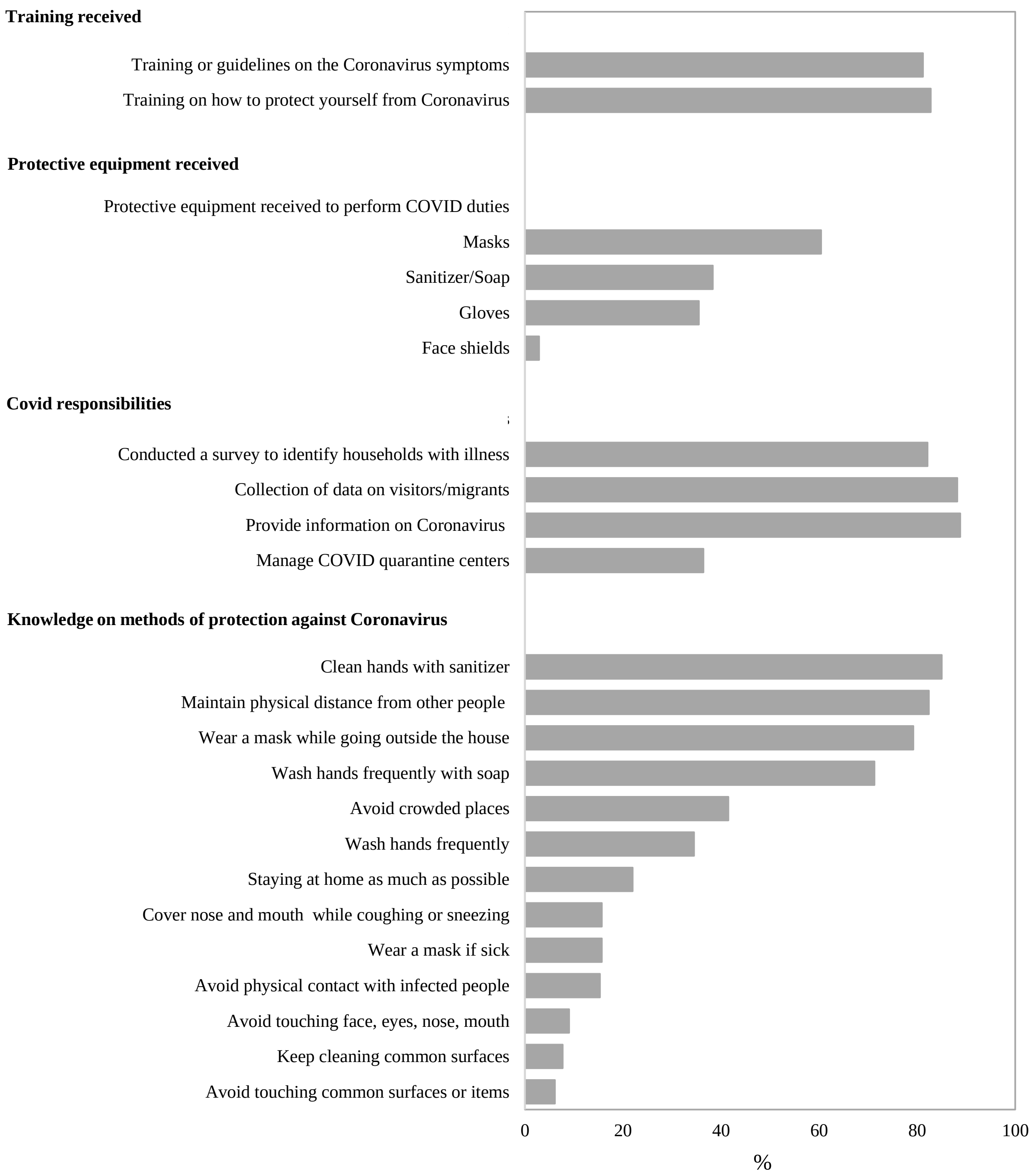

Supplement: nxab135_Supplemental_File [file nxab135_supplemental_file.pdf]
